# The Cheese Matrix Modulates the Immunomodulatory Properties of *Propionibacterium freudenreichii* CIRM-BIA 129 in Healthy Piglets

**DOI:** 10.3389/fmicb.2018.02584

**Published:** 2018-10-29

**Authors:** Houem Rabah, Stéphanie Ferret-Bernard, Song Huang, Laurence Le Normand, Fabien J. Cousin, Floriane Gaucher, Romain Jeantet, Gaëlle Boudry, Gwénaël Jan

**Affiliations:** ^1^STLO, INRA, Agrocampus Ouest, Rennes, France; ^2^Pôle Agronomique Ouest, Rennes, France; ^3^INRA, INSERM, Univ Rennes, Nutrition Metabolisms and Cancer, NuMeCan, Rennes, France; ^4^UNICAEN, UNIROUEN, ABTE, Normandie Université, Caen, France; ^5^Bioprox, Levallois-Perret, France

**Keywords:** probiotics, *P. freudenreichii*, delivery vehicle, cheese matrix, immunomodulation, T lymphocytes phenotype, PBMC, MLNCv

## Abstract

*Propionibacterium freudenreichii* is a beneficial bacterium, used as a cheese starter, which presents versatile probiotic properties. These properties are strain-dependent. We hypothesized they may also be delivery vehicle-dependent. In this study, we thus explored in healthy piglets how the cheese matrix affects the immunomodulatory properties of *P. freudenreichii*. During 2 weeks, three groups of weaned piglets consumed, respectively, *P. freudenreichii* as a liquid culture (PF-culture), *P. freudenreichii* under the form of a cheese (PF-cheese), or a control sterile cheese matrix (Cheese-matrix). The *in vivo* metabolic activity of *P. freudenreichii* was assessed by determining short chain fatty acids (SCFA) concentration and bifidobacteria population in feces. Whatever the delivery vehicle, *P. freudenreichii* was metabolically active in piglets’ colon and enhanced both bifidobacteria and SCFA in feces. *P. freudenreichii* consumption decreased the secretion of TNFα and of IL-10 by peripheral blood mononuclear cells (PBMC). It did not alter IL-10, IFNγ, IL-17, and TNFα secretion in mesenteric lymph node immune cells (MLNC). PF-cheese enhanced significantly Treg phenotype, while PF-culture decreased significantly Th17 phenotype in PBMC and MLNC. Remarkably, only PF-cheese induced an increase of Th2 phenotype in PBMC and MLNC. *Ex vivo* stimulation of PBMC and MLNC by Lipopolysaccharides and Concanavalin A emphasized the difference in the immunomodulatory responses between PF-culture and PF-cheese group, as well as between PBMC and MLNC. This study shows the importance to consider the delivery vehicle for probiotic administration. It confirms the anti-inflammatory potential of *P. freudenreichii*. It opens new perspectives for the use propionibacteria-fermented products as preventive agents for inflammatory bowel diseases and intestinal infectious diseases.

## Introduction

*Propionibacterium freudenreichii* is a beneficial bacterium, belonging to the Actinomycetales order. It has been recognized as safe (GRAS status) in the United States of America, and qualified presumption of safety (QPS status) in Europe. *P. freudenreichii* is a cheese starter used in Swiss-type cheeses manufacture such as Emmental. It moreover revealed versatile, strain-dependent, probiotic functionalities (Thierry et al., 2011; Rabah et al., 2017). These properties are strain-dependent and result from the production of several beneficial metabolites by propionibacteria (Cousin et al., 2011; Rabah et al., 2017). Short chain fatty acids (SCFA), especially propionic and acetic acids derived from lactate fermentation by propionibacteria, have anti-inflammatory and anti-cancerous effects on colonic intestinal cells (Jan et al., 2002; Lan et al., 2008; Cousin et al., 2012b, 2016). *P. freudenreichii* produces also bifidogenic factors, including 1,4-dihydroxy-2-naphtoic acid (DHNA),which enhances bifidobacteria growth and reduces inflammation in intestinal epithelial cells (Isawa et al., 2002; Okada, 2006; Suzuki et al., 2006; Rabah et al., 2017). Finally, the surface proteome of *P. freudenreichii* is involved in host-bacteria interaction, with a prominent role of non-covalently surface-bound proteins such as S-layer proteins (Slps) (Le Maréchal et al., 2015; Colliou et al., 2017; Deutsch et al., 2017; Do Carmo et al., 2018). The development of dairy fermented products as functional foods by screening specific starter bacteria which possess both probiotic and food fermentation abilities is a promising perspective. It will provide an alternative tool to prevent several inflammatory diseases, as inflammatory bowel diseases (IBD). A functional food is defined as an “ingredient that affects beneficially one or more target functions in the body, beyond adequate nutritional effects, in a way that is relevant to either an improved state of health and well-being and/or reduction of risk disease” (Saris et al., 1998; Diplock et al., 1999). Understanding how *P. freudenreichii* impacts health, specifically intestinal health, is crucial to develop functional dairy foods. Indeed, effects of dairy products, fermented solely by propionibacteria, or in combination with lactic acid bacteria, already revealed beneficial effects in a mice model of colitis (Plé et al., 2015, 2016; Foligné et al., 2016). The ingestion of these fermented dairy foods reduced the severity of chemically induced colitis. Recently, *P. freudenreichii* was shown to be part of the human milk microbiota and to participate in the prevention of necrotizing enterocolitis (NEC) in preterm infants (Colliou et al., 2017). These detailed studies highlighted the potential of *P. freudenreichii* consumption to prevent intestinal inflammatory diseases. However, investigations on the impact of the delivery vehicle on the probiotic functionalities of *P. freudenreichii* in healthy subjects are lacking. Here, we investigated the influence of the cheese matrix on *in vivo* probiotic functionalities of *P. freudenreichii*. Indeed, dairy matrices were shown to enhance propionibacteria tolerance toward digestive stresses, via overexpression of a panel of proteins involved in acid and bile salts stress responses (Leverrier et al., 2005; Saxelin et al., 2010; Gagnaire et al., 2015). In addition, the high concentration of dairy proteins in cheese plays a role as buffering agent toward gastric acids, in addition to the presence of lipids, which limits the toxic effect of bile salts on bacterial membranes (Rabah et al., 2017, 2018). Such tolerance may favor propionibacteria survival, enhance their metabolic activity, and consequently their immunomodulatory effects, within the gut. Furthermore, adhesion and immunomodulation, both mediated by S-layer proteins, may be promoted, since the cheese matrix protects these proteins from digestive proteolysis *in vitro* (Do Carmo et al., 2017; Rabah et al., 2018). In this study, we compared the effect of two delivery vehicles, a single-strain cheese (PF-cheese) and fresh culture in milk ultrafiltrate (PF-culture), in healthy piglets. Both delivery vehicles were fermented by the strain *P. freudenreichii* CIRM-BIA 129, which has been selected previously as the most anti-inflammatory one (Foligné et al., 2010, Foligné et al., 2013). The impact of the delivery vehicle on *in vivo* metabolic activity and on immunomodulation by *P. freudenreichii* was assessed in healthy piglets. Then, to seek a functional role of *P. freudenreichii* consumption by healthy animals against inflammation, we assessed piglets’ immune cell responses to exogenous proinflammatory stimulations.

## Materials and Methods

### Bacterial Strain and Dairy Matrices Preparation

The strain *P. freudenreichii* CIRM-BIA 129 (equivalent to ITGP20 strain) was provided by the French Dairy Interbranch Organization (Centre National Interprofessionnel de l’Economie Laitière, CNIEL) and maintained by the International Centre for Microbial Resources (Centre International de Ressources Microbiennes-Bactéries d’Intérêt Alimentaire, CIRM-BIA). Dairy propionibacteria were routinely cultivated at 30°C in yeast-extract-lactate medium (YEL). For the PF- culture, *P. freudenreichii* CIRM-BIA 129 was grown in milk ultrafiltrate supplemented with 100 mM sodium DL-lactate (50% in H_2_O, Sigma) and 5 g/L casein hydrolysate (Organotechnie, La Courneuve, France) (Cousin et al., 2012c) at 30°C, without agitation, in microaerophilic conditions until stationary phase (60 h of incubation). PF-cheese is a single-strain cheese fermented by *P. freudenreichii* CIRM-BIA 129 as described previously (Plé et al., 2015). The biochemical composition of the cheese was: dry matter 58 g/100 g, lipids 28 g/100 g, proteins 29 g/100 g, carbohydrates 0 g/100 g, and calcium 840 mg/100 g (Plé et al., 2015). The cheese matrix is a sterile dairy matrix prepared in the same way as the single strain cheese. Glucono delta-lactone was used to acidify the sterile supplemented milk before cheese matrix manufacturing procedure, as described previously (Plé et al., 2015). The propionibacteria amounts reached 5.10^9^ CFU/ml in PF-culture, and 1.10^10^ CFU/g in PF-cheese.

### Ethics Statement

The experimental protocol was performed in accordance with recommendations of the French law (2001-464 29/05/01) and EEC (86/609/CEE) for the care and use of laboratory animals. The protocol was approved by the ethical committee on animal experimentation of Rennes (France), under the certificate of authorization to experiment 2017010922379066-V2. Pigs were sacrificed by electronarcosis followed by exsanguination, and every effort was made to minimize animal suffering.

### Animal Procedures and Immune Cell Isolation

Twenty one [(Pietrain × Landrace) × (Large White)] 8-week old piglets (13.3 ± 0.4 kg) from the experimental herd of INRA St-Gilles (UEPR, France) were used. Three groups of seven piglets were constituted: (1) Cheese matrix (10 g), (2) PF-culture (1.10^11^ CFU of *P. freudenreichii*) and (3) PF-cheese (1.10^11^ CFU of *P. freudenreichii*). PF-cheese and cheese matrix were mixed by a turrax (Ultra-turrax T8 IKA, Fischer Scientific, 20,000 tr/min-2 min) in four volumes of sterile physiological water. Piglets were gavaged using syringes every morning (between 9.00 and 10.00 am) during 14 days. They were fed with a standard pig diet that was given at 10.00 am. Food was removed from the cage at 4:00 pm to monitor daily food intake. Animals were fasted from 4:00 pm to 9:00 am but had free access to water. Piglets were weighed five times (d0, d1, d4, d7, d10, and d14) and fecal samples were collected at day 0, day 7, and day 14. At the end of the 14-day treatment period, pigs were sacrificed 30 min after their last gavage by electronarcosis then exsanguination. Blood was collected in sterile BD vacutainer^®^CPT^TM^ tubes (containing sodium heparin as well as Ficoll^TM^Hypaque^TM^ density fluid and a polyester gel barrier) at room temperature. Following centrifugation at 1500 *g* for 20 min without brake, we isolated the peripheral blood mononuclear cells (PBMC) by carefully pipetting the interface above the gel barrier. Additional washing in Hank’s balanced saline solution (HBSS), supplemented with 200 UI/ml penicillin and 200 μg/ml streptomycin, and centrifugation steps resulted in a suspension of concentrated mononuclear cells. After laparotomy, 4 g of intestinal mesenteric lymph nodes (MLN) were placed in ice-cold HBSS for mononuclear cell isolation, as already described (Ferret-Bernard et al., 2010).

### Quantification of Fecal Propionibacteria, Lactobacilli and Bifidobacteria Populations

Propionibacteria, Lactobacilli, and Bifidobacteria were quantified in feces collected at day 0 (before treatment) and day 14 (end of the treatment). Samples were analyzed in duplicate. For propionibacteria quantification, QIAamp DNA Stool Kit was used to extract DNA, as described previously (Hervé et al., 2007; Cousin et al., 2012a). For lactobacilli and bifidobacteria quantification, DNeasy Blood & Tissue Kits (Qiagen) was used to extract DNA from pure cultures of *Bifidobacterium longum* CIRM-BIA 1336 or *Lactobacillus pentosus* CIRM-BIA 660. Propionibacteria concentrations were measured by qPCR of 5S subunit gene of transcarboxylase as described previously (Supplementary Table S1) (Hervé et al., 2007; Cousin et al., 2012a). Briefly, ten-fold dilutions of the *P. freudenreichii* CIRM-BIA 129 were prepared in saline solution and enumerated using YELA medium. One hundred microliters of each dilution was then added to 200 mg of feces from naive pig (exempt of propionibacteria) and thoroughly mixed. DNA was then extracted in the same way as unknown samples. A standard curve was generated and results are expressed as log [bacteria] per gram of sample. For lactobacilli and bifidobacteria quantification, 10-fold serial dilutions of target genomic DNA extracted from pure cultures of *B. longum* CIRM-BIA 1336 or *L. pentosus* CIRM-BIA 660 were performed (Supplementary Table S1). The linear equation for the standard curve was then used to interpolate the numbers of copies present in the unknown samples.

### Short Chain Fatty Acids Analysis

Short chain fatty acid concentration was determined in fecal samples at days 7 and 14. Immediately after collection, fecal samples were diluted in ortho-phosphoric acid (50% V/V) to stop fermentation and samples were stored at −20°C until analysis. SCFA were separated on a BP20 (SGE) column and quantified by a flame ionization detector as previously described (Jouany et al., 1981). Isocaproic acid was used as an internal standard. Samples were analyzed in duplicate, and the results are expressed as micromolar per g of feces.

### PBMC and MLNC Stimulation

Immune cells were suspended in complete RPMI 1640 medium (Sigma) supplemented with 10% fetal calf serum (FCS), 100 IU/ml penicillin and 100 mg/ml streptomycin to achieve cell concentration of 5 × 10^6^ cells/ml for PBMC and 10 × 10^6^ cells/ml for MLNC in 96-well flat-bottomed plates. Cells were stimulated for 72 h at 37°C, under an atmosphere containing 5% CO_2_, in unstimulated condition (complete RPMI alone) or in presence of 200 μg/ml of *P. freudenreichii*’ S-layer proteins (Slps). Slps were extracted (Le Maréchal et al., 2015), partially purified by size exclusion chromatography as previously described (De sa Peixoto et al., 2015) and proteins were concentrated by filtration using VivaSpin-10 kDa. PBMC and MLNC were also cultivated in presence of 10 μg/ml of Lipopolysaccharides (LPS) from *Escherichia coli* 0111:B4, or in presence of 0.5 μg/ml of Concanavalin A (ConA, sub-optimal concentration), or a combination of Slps + LPS and Slps + ConA. Culture supernatants of PBMC and MLNC were harvested and stored at −20°C until assayed for cytokine detection. Remaining cells were re-suspended in FCS 10% DMSO (Hybri-max, Sigma) and stored at −150°C until mRNA extraction.

### Cytokine Patterns of PBMC and MLNC

Concentrations of IL-10, IFNγ, and TNFα were measured in culture supernatants of PBMC and MLNC, using capture sandwich ELISA porcine ELISA kit (R&D Systems, Lille, France) according to the manufacturer’s instructions. IL-17 concentration was measured also using capture sandwich swine ELISA kit (VetSet^TM^, Kingfisher Biotech, United States). Cytokine concentrations after stimulation were given in pictograms per ml of supernatant.

### RT-qPCR

Quantitative PCR was performed to determine *Tbet*, *GATA3*, *FOXP3*, and *ROR*γ*t* mRNA levels in PBMC and MLNC. Primers used for mRNA quantification are listed in Supplementary Table S1. Total RNA from cells was isolated by Trizol reagent (Invitrogen Ambion), and cDNA was synthesized using a qScript cDNA synthesis kit (Quanta Biosciences). Amplification was performed as previously described (Rabah et al., 2018). The transcripts level of the target genes was normalized to the transcript level of *hprt* gene (Ledger et al., 2004) (housekeeping gene, see Supplementary Table S1). These primers were described previously (Muráni et al., 2007; Delroisse et al., 2008; Hernández et al., 2009; Young et al., 2012; Hermann-Bank et al., 2013; Zhu et al., 2014). *Hprt* expression was not affected by the tested matrices. The results are expressed as expression level (2^−ΔCT^), in duplicate analysis for each piglets (*n* = 7) for the three groups.

### Statistical Analysis

We analyzed all data with non-parametric tests after checking the non-Gaussian distribution of data. The effect the consumption of *P. freudenreichii* on the fecal propionibacteria amounts was analyzed with the Mann–Whitney test by comparing only PF-culture and PF-cheese groups. The difference in lactobacilli and bifidobacteria was analyzed separately by comparing the concentration at day 0 to the concentrations determined at day 14 using non-parametric ANOVA with Dunn’s multiple comparison test as a *post hoc* test. The same test was also performed to compare the three groups in term of SCFA concentration, basal cytokine concentrations and gene expression. To analyze cytokine secretion after ConA and LPS stimulation, 2-way non-parametric ANOVA was performed with the sidak’ s multiple comparison test as a *post hoc* test. Statistical significance was set at *p* < 0.05. Calculations were performed using GraphPad Prism Software (Prism 7 for Windows). All data were expressed as mean values and standard error of the mean (SEM) (*n* = 7).

## Results

### Piglet Growth and Food Intake

Food intake was similar among the three experimental groups (Supplementary Figure S1A). Piglets had a similar growth without significant difference between experimental groups (Supplementary Figure S1B). Feed efficiency was also similar among experimental groups (Supplementary Figure S1C). No health problems was encountered during the 14-day experimental period.

### Quantification of Fecal Population of Propionibacteria, Bifidobacteria, and Lactobacilli

**FIGURE 1:**
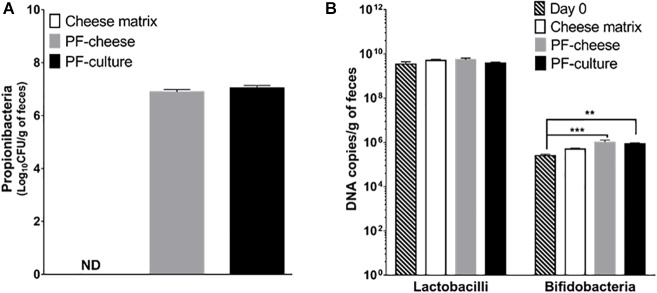
Propionibacteria total population **(A)**, lactobacilli and bifidobacteria **(B)** total population in feces were assessed at day 14 in the different piglets groups: cheese matrix, PF-culture or PF-cheese fermented both by *P. freudenreichii* CIRM-BIA 129 (PF). Propionibacteria, lactobacilli, and bifidobacteria concentrations were determined by qPCR in feces. Results are represented as means ± SEM (*n* = 7). ^∗∗^*P* < 0.005 and ^∗∗∗^*P* < 0.0005.

Fecal propionibacteria population, as determined by qPCR, was undetectable before treatment (day 0, data not shown). Propionibacteria remained also undetectable in the cheese matrix piglets group’s feces (Figure 1A). After 2 weeks of gavage, propionibacteria reached 7.0 ± 0.1 log/g of feces in the PF-culture piglets and 6.9 ± 0.1 log/g of feces in the PF-cheese piglets, with no significant difference between the two groups (*p* > 0.05). The impact of *P. freudenreichii* consumption on piglet’s microbiota was investigated, focusing on two genera, using qPCR. Lactobacilli and bifidobacteria populations between the different treated groups at day 14 were compared to the population level at day 0. *P. freudenreichii* ingestion significantly enhanced bifidobacteria in feces (Figure 1B). By contrast, none of the treatments induced significant change in the population of lactobacilli, regardless of the delivery vehicle (Figure 1B).

### SCFA Quantification in Piglet Feces

**FIGURE 2:**
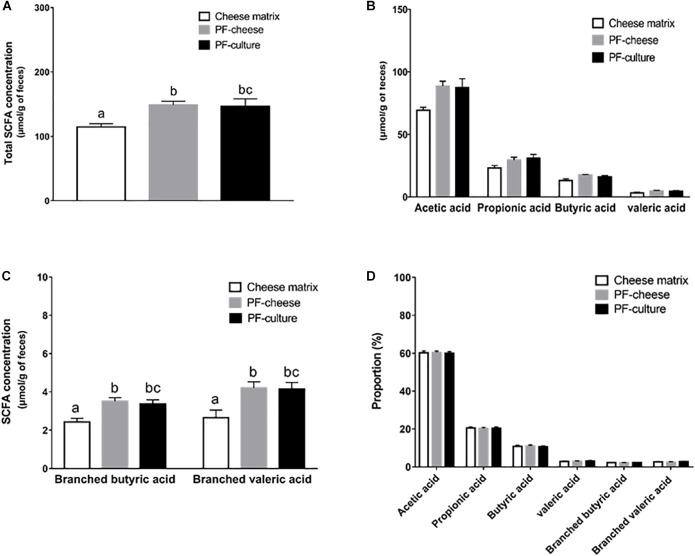
Analysis of short chain fatty acids (SCFA) concentration in feces contents at day 14 of the three piglets groups fed with: cheese matrix, PF-culture or PF-cheese fermented by *P. freudenreichii* CIRM-BIA 129 (PF). The total SCFA concentration **(A)**, the concentration of each SCFA type (C2, C3, C4, and C5) **(B)**, and the concentration of branched short chain fatty acids **(C)** were measured by gas-phase chromatography, thus the proportion of each type of SCFA **(D)** was determined. Results are represented as means ± SEM (*n* = 7). Letters denotes significant difference, *P* < 0.05.

To assess the *in vivo* metabolic activity of *P. freudenreichii*, SCFA concentration were determined in feces at days 7 and 14. At day 7, SCFA concentrations in feces were equivalent among the different groups (Supplementary Figure S2). However, at day 14, *P. freudenreichii* consumption enhanced significantly total SCFA concentration in feces, regardless of the delivery matrix (PF-culture or PF-cheese, *p* = 0.98) (Figure 2A). *P. freudenreichii* consumption tended to increase acetic acid concentration, compared to the cheese matrix group (*p* = 0.062 for PF-culture group and *p* = 0.069 for PF-cheese group) (Figure 2B). By contrast, propionic, butyric or valeric acid concentrations were not significantly enhanced (Figure 2B). Regarding the concentrations of branched chain fatty acids (BCFA), PF-culture piglets and PF-cheese piglets displayed increased fecal branched valeric and butyric acids concentration, compared to cheese matrix group (Figure 2C). In addition, compared to cheese matrix group, no significant difference in BCFA concentrations between the PF-culture and the PF-cheese groups were observed (Figure 2C). The proportions of the different SCFA were not modified by the experimental treatments (Figure 2D).

### Anti-inflammatory Properties of *P. freudenreichii* Slps Proteins on Naive Swine PBMC and MLNC

**FIGURE 3:**
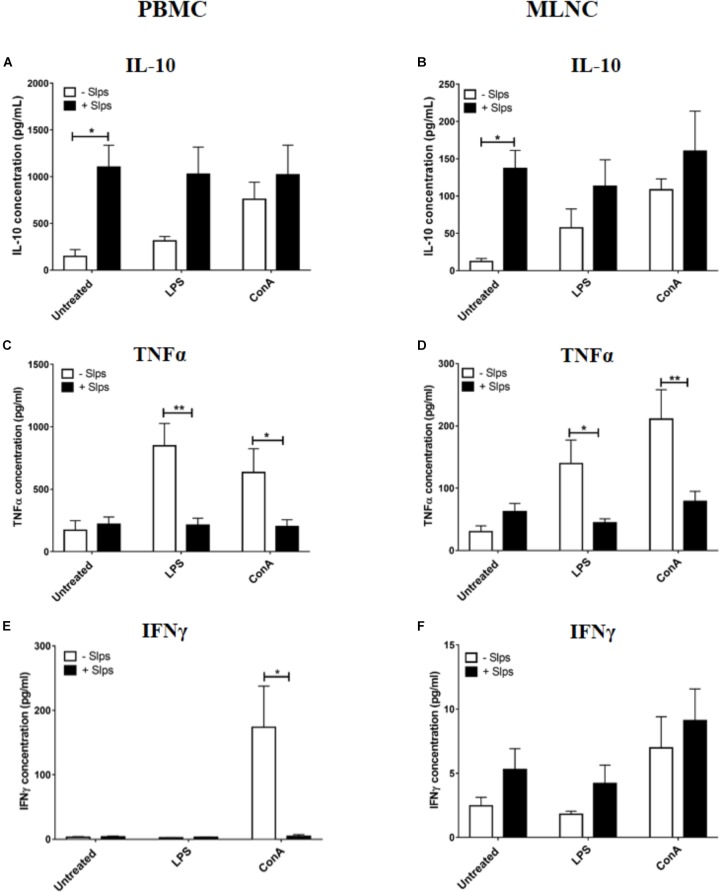
Secretion of **(A,B)** IL-10, **(C,D)** TNFα, and **(E,F)** IFNγ by peripheral blood mononuclear cells (PBMC) and mesenteric lymph nodes cells (MLNC) from naive piglets (cheese matrix group). Cells were stimulated *ex vivo* with Lipopolysaccharides (LPS), Concanavalin A (ConA) in combination with *P. freudenreichii*’ S-layer proteins (Slps). Cytokine concentrations were measured by ELISA and represented as means ± SEM (*n* = 7). ^∗^*P* < 0.05 and ^∗∗^*P* < 0.01.

To verify the effect of *P. freudenreichii* Slps on naive swine immune cells as already demonstrated in other species (Le Maréchal et al., 2015), PBMC and MLNC from cheese matrix piglets were stimulated by Slps in the presence of LPS or ConA. Slps significantly increased IL-10 secretion by both PBMC and MLNC (Figures 3A,B). In the presence of ConA, Slps did not influence secretion of IL-10 by both cells types (Figures 3A,B). In the presence of LPS, Slps tended to increase IL-10 secretion by PBMC (*p* = 0.0763) (Figure 3A). Slps significantly reduced LPS or ConA-induced TNFα secretion by PBMC and MLNC (Figures 3C,D). Only ConA triggered a high secretion of IFNγ by PBMC, which was significantly reduced by the co-stimulation with Slps (Figures 3E,F).

### Basal Cytokines Secretion by PBMC and MLNC

**FIGURE 4:**
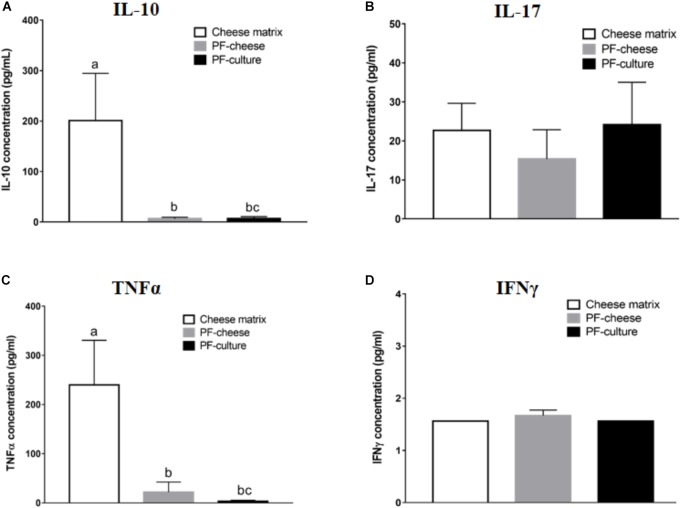
Basal secretion of **(A)** IL-10, **(B)** IL-17, **(C)** TNFα, and **(D)** IFNγ by PBMC of different piglets groups fed with: cheese matrix, PF-culture or PF-cheese both fermented by *P. freudenreichii* CIRM-BIA 129 (PF). Cytokine concentrations were determined by ELISA and represented as means ± SEM (*n* = 7). Letters denotes significant difference, *P* < 0.05.

*Propionibacterium freudenreichii* consumption decreased basal secretion of IL-10 by PBMC, compared to cheese matrix group, whatever the delivery vehicle (Figure 4A). Basal TNFα secretion by PBMCs was significantly lower in PF-culture and PF-cheese groups, compared to cheese matrix group (Figure 4C). Finally, basal secretion of IFNγ and IL-17 by PBMC was similar in all piglet groups (Figures 4B,D). By contrast, in MLNC, IL-10, TNFα, IL-17, and IFNγ secretion were similar among the different experimental groups (Supplementary Figures S3A–D).

### T Lymphocytes Phenotype in PBMC and MLNC

**FIGURE 5:**
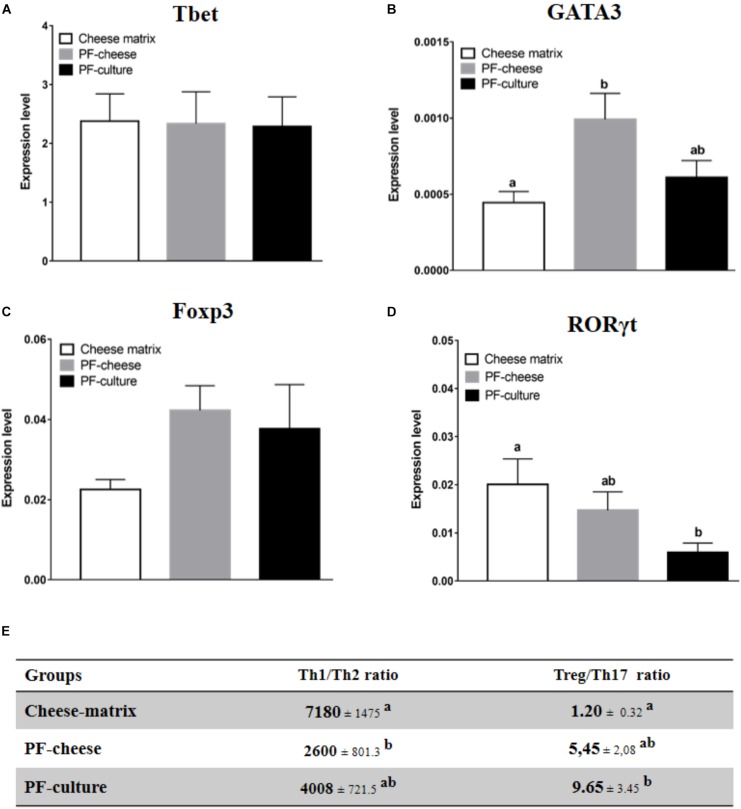
Basal expression of **(A)** Tbet, **(B)** GATA3, **(C)** FOXP3, and **(D)** in PBMC from different piglets groups fed with cheese matrix, PF-culture or PF-cheese both fermented by *P. freudenreichii* CIRM-BIA 129 (PF). The level of expression was determined by RT-PCR. Ratios of Th1/Th2 and Treg/Th17 **(E)** were determined by calculating, respectively, Tbet/GATA3 and Foxp3/RORγt ratios. Results are represented as means ± SEM (*n* = 7). Letters denotes significant difference, *P* < 0.05.

Phenotype of T lymphocytes population in PBMC and MLNC was assessed by analyzing the expression of transcriptions factors: Tbet, GATA3, Foxp3, and RORγt, respectively, specific of Th1, Th2, Treg, and Th17 lymphocytes populations. Tbet expression in PBMC was similar among the different piglets groups (Figure 5A). PF-cheese, but not PF-culture consumption, increased significantly GATA3 expression in PBMC compared to the cheese matrix group (Figure 5B). The Th1/Th2 ratio was significantly lower in PF-cheese piglets, compared to cheese matrix ones, without significant difference with PF-culture piglets (Figure 5E). PBMC from PF-cheese piglets tended to express more Foxp3, compared to cheese matrix ones (*p* = 0.0636) (Figure 5C). PBMC from PF-culture piglets, but not from PF-cheese ones, had a significantly lower expression of RORγt compared to cheese-matrix piglets (Figure 5D). By determining Treg/Th17 ratio, we observed that only PF-culture piglets displayed a significant increase of Treg/Th17 ratio, compared to the cheese matrix piglets (Figure 5E). The Treg/Th17 ratio tended also to be higher in PF-cheese piglets, compared to cheese matrix ones (*p* = 0.0666) (Figure 5E).

**FIGURE 6:**
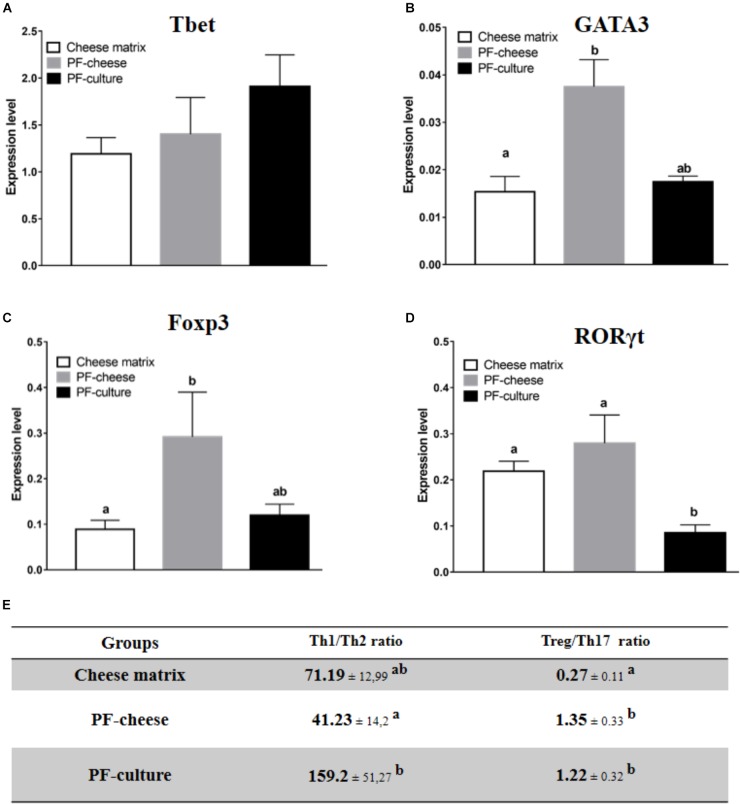
Basal expression of **(A)** Tbet, **(B)** GATA3, **(C)** FOXP3, and **(D)** RORγt were analyzed in MLNC from different piglets groups fed with: cheese matrix, PF-culture or PF-cheese both fermented by *P. freudenreichii* CIRM-BIA 129 (PF). The level expression was determined by RT-PCR. Ratios of Th1/Th2 and Treg/Th17 **(E)** were determined by calculating, respectively, Tbet/GATA3 and Foxp3/RORγt ratios. Results are represented as means ± SEM (*n* = 7). Letters denotes significant difference, *P* < 0.05.

Tbet expression was similar in MLNC from the different piglet groups (Figure 6A). Consumption of PF-cheese significantly increased GATA3 expression in MLNC, compared to the cheese matrix group, but without significant difference compared with PF-culture group (Figure 6B). MLNC from PF-cheese piglets displayed greater Foxp3 expression, compared to MLNC from both cheese matrix and PF-culture groups (Figure 6C). Consumption of PF-cheese significantly decreased Th1/Th2 ratio, compared to PF-culture group, but without significant difference with cheese matrix group. RORγt expression was lower in MLNC from PF-culture piglets compared to PF-cheese and cheese matrix groups (Figure 6D). Consumption of *P. freudenreichii*, regardless of the delivery vehicle, significantly increased the Treg/Th17 ratio (Figure 6E).

### Cytokine Secretion by PBMC and MLNC in Response to *ex vivo* Stimuli

**FIGURE 7:**
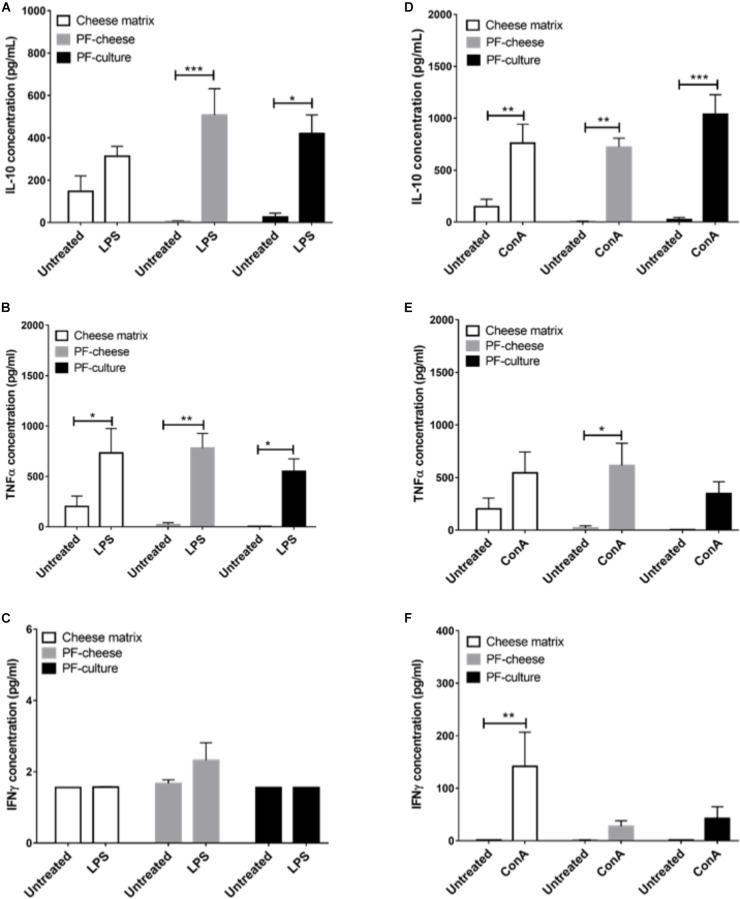
Secretion of IL-10, TNFα, and IFNγ of stimulated PBMC from different piglets groups fed with: cheese matrix, PF-culture or PF-cheese fermented by *P. freudenreichii* CIRM-BIA 129 (PF). Cells were stimulated ex vivo with **(A–C)** LPS or **(D–F)** Concanavalin A (ConA). Cytokine concentrations were measured by ELISA and represented as means ± SEM (*n* = 7). ^∗^*P* < 0.05, ^∗∗^*P* < 0.005, and ^∗∗∗^*P* < 0.001.

The LPS stimulation of PBMC induced a significant increase of IL-10 secretion, compared to untreated cells, only by PBMC from PF-culture and PF-cheese groups (Figure 7A). LPS stimulation induced also a significant increase of TNFα secretion by PBMC, compared to untreated cells, in all groups (Figure 7B). However, IFNγ secretion by PBMC was not triggered by LPS in any group (Figure 7C). ConA stimulation of PBMC induced a significant increase of IL-10 secretion compared to untreated cells, with no difference between groups (Figure 7D). ConA stimulation triggered a significant increase of TNFα secretion compared to untreated cells, only in PBMC from PF-cheese group. PBMC from PF-culture group tended also (*p* = 0.0676) to secrete more TNFα in response to ConA stimulation (Figure 7E). Stimulation by ConA induced a significant increase of IFNγ secretion, compared to untreated cells, only in PBMC from cheese matrix group (Figure 7F).

**FIGURE 8:**
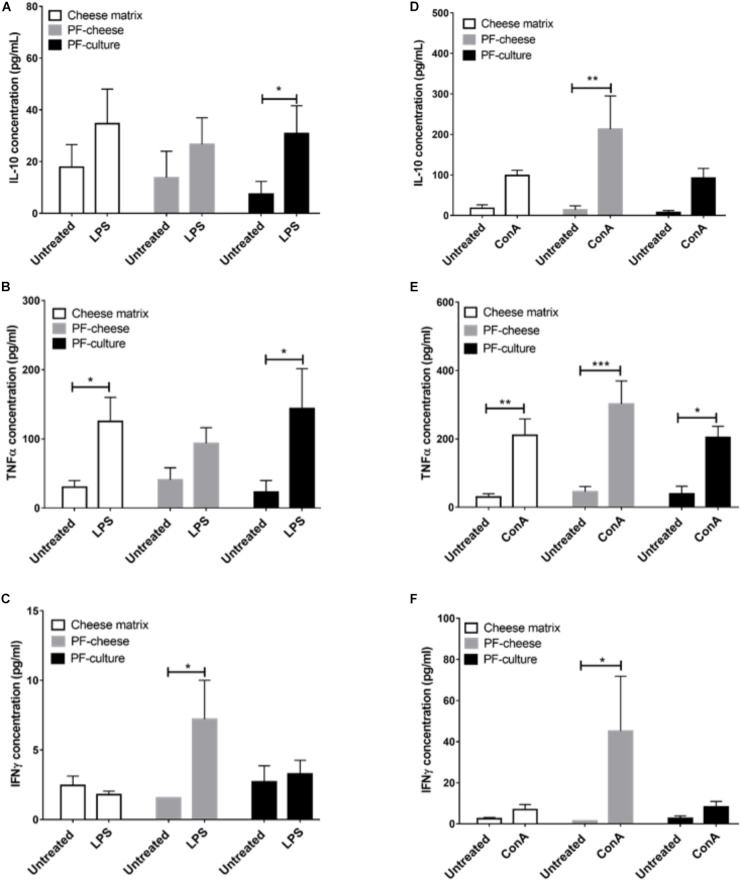
Secretion of IL-10, TNFα, and IFNγ of stimulated MLNC from different piglets groups fed with: cheese matrix, PF-culture or PF-cheese fermented by *P. freudenreichii* CIRM-BIA 129 (PF). Cells were stimulated ex vivo with **(A–C)** Lipopolysaccharides (LPS) or **(D–F)** Concanavalin A (ConA). Cytokine concentrations were measured by ELISA and represented as means ± SEM (*n* = 7). ^∗^*P* < 0.05, ^∗∗^*P* < 0.005, ^∗∗∗^*P* < 0.001.

The LPS Stimulation of MLNC increased significantly IL-10 secretion compared to untreated cells, only in MLNC from PF-culture group (Figure 8A). It induced also a significant increase of TNFα secretion by MLNC from cheese matrix and PF-culture piglets but not PF-cheese ones (Figure 8B). In addition, LPS stimulation induced a slight but significant secretion of IFNγ compared to untreated cells, only in MLNC from PF-cheese group (Figure 8C).

ConA triggered a significant rise of IL-10 secretion only in MLNC from PF-cheese group (Figure 8D). ConA stimulation of MLNC from all groups induced a significant secretion of TNFα compared to untreated cells (Figure 8E). Similarly, ConA stimulation significantly increased IFNγ secretion compared to untreated cells, only in MLNC from PF-cheese piglets (Figure 8F).

## Discussion

The aim of this study was to investigate the impact of the delivery vehicle on the probiotic functionalities of *P. freudenreichii* in healthy piglets. For this purpose, three groups of piglets were fed during 2 weeks, with sterile cheese matrix, or a fresh culture of *P. freudenreichii* (PF-culture, 10^11^ CFU/day) or a single-strain cheese (PF-cheese, 10^11^ CFU/day) fermented solely by *P. freudenreichii* CIRM-BIA 129.

We investigated firstly if *P. freudenreichii* was metabolically active in piglet colon. *P. freudenreichii* metabolically active enhanced SCFA concentration in rats (Lan et al., 2007) and increased bifidobacteria in humans’ fecal contents (Bouglé et al., 1999). The ability of a probiotic to be metabolically active in the colon depends on its adaptability toward colonic environment. *In vitro* studies suggested that propionibacteria digestive stresses tolerance is strain-dependent (Leverrier et al., 2005 ; Cousin et al., 2012c), but also matrix-dependent (Leverrier et al., 2005; Cousin et al., 2012c; Huang et al., 2016; Rabah et al., 2018). By contrast, our study demonstrated that both vehicles delivered metabolically active *P. freudenreichii* to the piglets’ colon. To attest of the *in vivo* metabolic activity of *P. freudenreichii*, we investigated its bifidogenic effect. Indeed, numerous studies demonstrated that *P. freudenreichii* has a bifidogenic effect (Bouglé et al., 1999; Hojo et al., 2002; Colliou et al., 2017) due to the *in situ* production of several metabolites (Yamazaki et al., 1999; Isawa et al., 2002; Kouya et al., 2007). *P. freudenreichii* consumption enhanced bifidobacteria population, without affecting lactobacilli population, in fecal contents, compared to cheese matrix group. *P. freudenreichii* metabolic activity was also assessed by analyzing fecal SCFA concentration. In our study, *P. freudenreichii* CIRM-BIA 129 significantly increased total SCFA concentration, in particularly BCFA concentrations. The ability of *P. freudenreichii* to produce BCFA has already been demonstrated (Dherbécourt et al., 2008). These results are consistent with a previous transcriptomic study demonstrating that *P. freudenreichii*, within the colon, represses the wood-werkman cycle pathway (suggesting decreased propionic and acetic acid production), while inducing branched-chain amino acid degradation (suggesting an increase of BCFA production) (Saraoui et al., 2013). The modulation of SCFA content could also result indirectly from the increase of bifidobacteria population (Ríos-Covián et al., 2016; Hemalatha et al., 2017; LeBlanc et al., 2017). In a previous study, *P. freudenreichii* CIRM-BIA 129, with a dose of 1.10^10^ CFU/day, did not change colonic SCFA concentration in healthy piglets (Cousin et al., 2012a). However, in our study, piglets receiving 10^11^ CFU of *P. freudenreichii* per day showed an increase of fecal SCFA concentration. These results suggest the major importance of the dose of live propionibacteria when seeking *in vivo* beneficial metabolic effects.

We examined *ex vivo* the immunomodulatory effect of extracted *P. freudenreichii* Slps on PBMC and MLNC from naive piglets (cheese matrix group). As already demonstrated with human PBMC (Le Maréchal et al., 2015; Deutsch et al., 2017), Slps induced high IL-10 secretion in swine PBMC. We further extended this observation to swine MLNC. In addition, Slps inhibited the secretion of TNFα induced by LPS and ConA stimulation, in both cell types. Slps also inhibited ConA-induced IFNγ secretion in PBMC. Indeed, these results are consistent with a previous study, showing that *P. freudenreichii* CIRM-BIA 129 Slps decreased IFNγ induced by the pro-inflammatory *Lactococcus lactis* MG1363 strain in human PBMC (Le Maréchal et al., 2015). The molecular mechanisms by which propionibacteria Slps suppress induction of pro-inflammatory cytokines triggered by ConA and LPS remain unexplained. Few studies showed the ability of Slps from *P. freudenreichii* (Colliou et al., 2017) or from other probiotics (Konstantinov et al., 2008; Martínez et al., 2012; Prado Acosta et al., 2016) to bind to C-type lectins receptors of monocytes. More investigations are, however, needed to elucidate the detailed molecular mechanism.

To explore the immunomodulatory effects of *P. freudenreichii* consumption at the systemic level, immune cells from blood (PBMC) were isolated. At the basal state, cytokines secretion and T lymphocytes phenotypes were analyzed. *P. freudenreichii* consumption, whatever the delivery vehicle, modulated Treg/Th17 ratio, compared to cheese matrix group. Nevertheless, PF-culture decreased significantly Th17 phenotype, compared to cheese matrix group. Contrastingly, PF-cheese tended to enhance Treg phenotype, compared to the cheese matrix group. In addition, only PF-cheese significantly modulated PBMC Th1/Th2 ratio toward a Th2 phenotype. However, basal cytokine secretions were not consistent with T lymphocytes PBMC phenotypes investigated by qPCR. *P. freudenreichii* consumption, in both delivery vehicles, decreased basal PBMC secretion of IL-10 (a cytokine produced by Treg and Th2 cells) and of TNFα (a cytokine produced by Th1 cells), compared to cheese matrix group. Basal cytokine secretion is a global response of T, B cells and innate immune cells (macrophages and dendritic cells), which may explain these discrepancies. Moreover, we did not evaluate IL-4 basal secretion, a marker of Th2 T cells. Finally *in vitro* studies showed *P. freudenreichii* CIRM-BIA 129 as an inducer of IL-10 and TNFα in human PBMC (Kekkonen et al., 2008; Le Maréchal et al., 2015; Deutsch et al., 2017), which is in contradiction to the *in vivo* results obtained in this study. This result suggests that chronic ingestion of *P. freudenreichii* affects differently immune cells than *ex vivo* acute stimulation. Noteworthy, *P. freudenreichii* consumption inhibited IFNγ secretion by PBMC in response to ConA stimulation, regardless of the delivery vehicle. Further research is needed to understand this delivery vehicle-induced switch in TNFα/IFNγ secretion.

The intestinal immune response to *P. freudenreichii* consumption was also investigated. *P. freudenreichii*, regardless of the delivery vehicle, did not affect basal secretion of IL-10, IL-17, TNFα, or IFNγ. Once again, cytokine secretion patterns were not consistent with the phenotype of T lymphocytes in the different piglet groups. The effects of *P. freudenreichii* on T lymphocytes phenotypes of MLNC were similar to that of PBMC. PF-cheese enhanced Treg phenotype and PF-culture decreased Th17 phenotype, compared to cheese matrix group. Moreover, PF-cheese enhanced Th2 phenotype, compared to cheese matrix group, but without significant difference with the PF-culture group. MLNC responses to LPS and ConA stimulation showed different responses between PF-cheese and PF-culture groups from that of PBMC. Indeed, in response to LPS stimulation, PF-culture consumption enhanced IL-10 and TNFα secretion by MLNC, compared to untreated cells, while PF-cheese consumption induced a slight, yet significant increase in IFNγ secretion. In addition, only MLNC from PF-cheese group showed a high secretion of IL-10 and IFNγ, compared to untreated cells. ConA in naive MLNC did not induce secretion of IFNγ. Moreover, there was no significant difference between the three groups in Th1 phenotypes, an IFNγ-secreting phenotype (Takaoka and Yanai, 2006; Kak et al., 2018). IFNγ may be also secreted by others innate immune cells (Takaoka and Yanai, 2006; Kak et al., 2018). This result suggests that PF-cheese modulates innate immune cells differently from PF-culture. IFNγ plays a primordial role in immune cells to fight intestinal infections: it activates macrophages and Th1 expansion, which induces an effective immune response against pathogens (Kak et al., 2018). In addition, only PF-cheese enhanced Th2 phenotype. Thus, the secretion of IFNγ may be a way to control Th2 cells expansion. These results suggest the potential of using *P. freudenreichii* CIRM-BIA 129 in functional foods to prevent intestinal infections, as already shown with *Listeria monocytogenes* infection in mice model (Kato-Mori et al., 2010; Colliou et al., 2018). This should be confirmed in the future by *in vivo* pathogens challenge experiments in piglets.

Altogether, *P. freudenreichii* showed an anti-inflammatory effect on the systemic and intestinal immune system by enhancing Treg and Th2 phenotypes or decreasing Th17 phenotype, depending on the delivery vehicle. Th2 and Treg responses triggered by *B. breve* was shown to protect mice from chemically induced colitis (Zheng et al., 2014). This may partially explain the protective role of *P. freudenreichii* in cheese against colitis (Plé et al., 2015, 2016; Foligné et al., 2016). Previously, *P. freudenreichii* P.UF1 was shown to increase Th17 population, to sustain Treg population and to decrease Th1 population, via a S-layer protein, in mice (Colliou et al., 2017, 2018).

In this study, since a similar metabolic activity of *P. freudenreichii* between the two delivery vehicles was observed, we assumed also that the matrix-dependent immunomodulatory effect may be related to surface proteins that would be protected by the cheese matrix (Rabah et al., 2018). More investigations are needed to confirm this hypothesis and to elucidate the molecular mechanism triggered by *P. freudenreichii* CIRM-BIA 129 to interact with host immune system.

## Conclusion

The present study demonstrated that *P. freudenreichii* exerts an anti-inflammatory effect, regardless of the delivery vehicle. The difference between vehicles in term of immunomodulatory modulation was obvious after *ex vivo* stimulation of immunes cells by LPS and ConA, in particularly at the intestinal level. Our study shows that the delivery vehicle should be carefully considered. It opens the perspective to use *P. freudenreichii* in cheeses to prevent IBD or intestinal infectious diseases.

## Author Contributions

GB, GJ, and SF-B designed the study. HR and SH prepared all matrices. HR, SH, FG, RJ, SF-B, LLN, GB, and GJ participated in animal experiment and laboratory analyses. HR and GB analyzed data and prepared figures. HR wrote the manuscript with the help of GB, FC, SF-B, and GJ. RJ, GB, and GJ supervised the project. All authors read and approved the final manuscript.

## Conflict of Interest Statement

FG was employed by company Bioprox. The remaining authors declare that the research was conducted in the absence of any commercial or financial relationships that could be construed as a potential conflict of interest.
