# Supplementary figures and images for: The Cheese Matrix Modulates the Immunomodulatory Properties of Propionibacterium freudenreichii CIRM-BIA 129 in Healthy Piglets
Source: Front Microbiol. 2018 Oct 29;9:2584. doi: 10.3389/fmicb.2018.02584 (PMC6215859; doi:10.3389/fmicb.2018.02584)

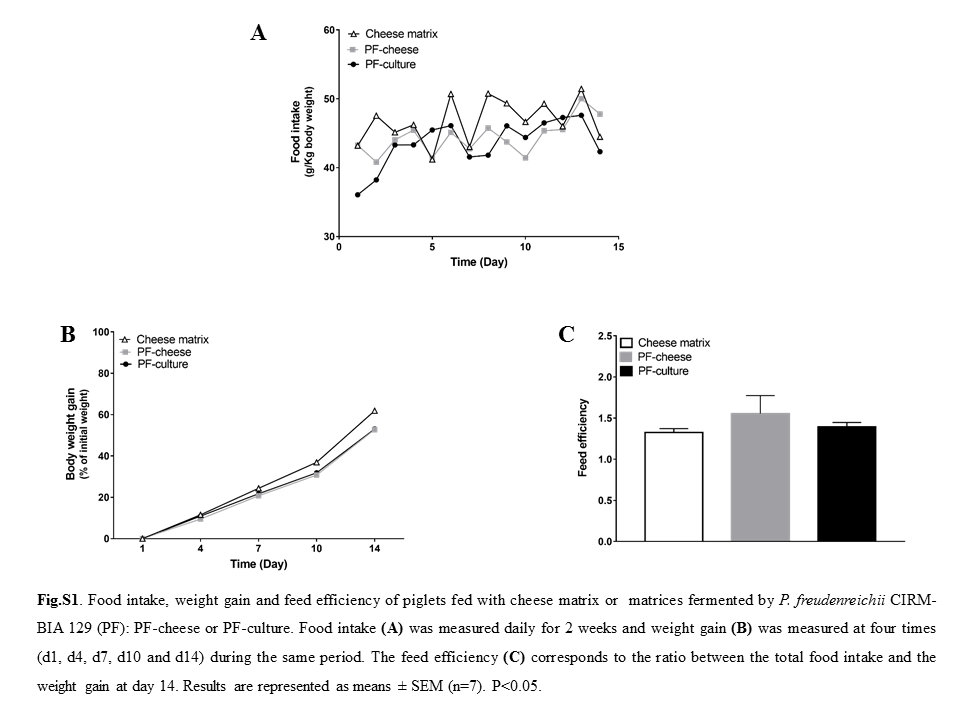

Supplement: Supplementary file 1 [file Image_1.TIF]

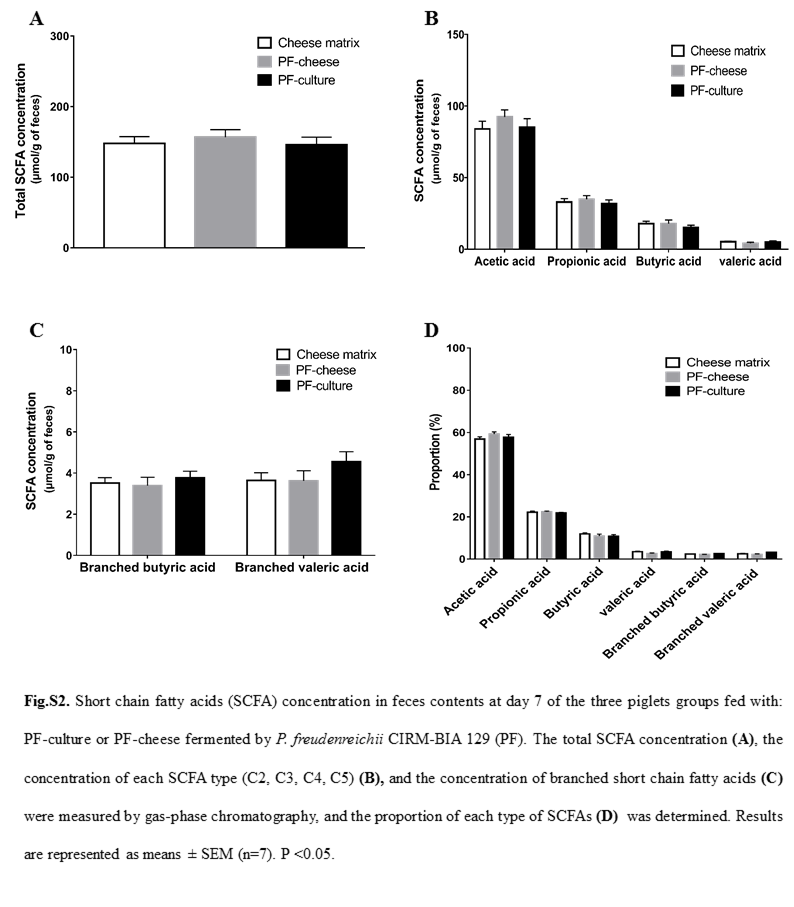

Supplement: Supplementary file 2 [file Image_2.tif]

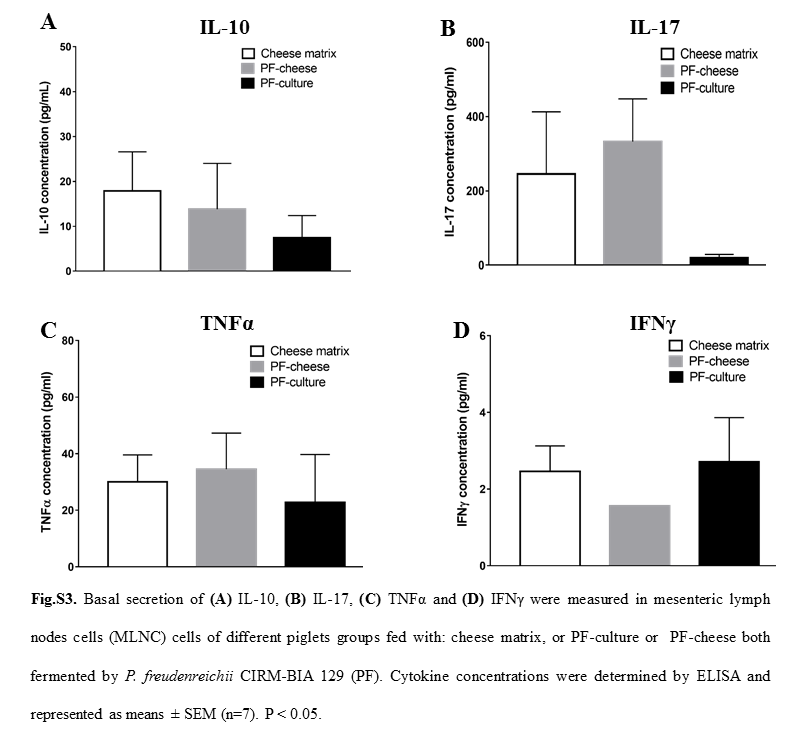

Supplement: Supplementary file 3 [file Image_3.tif]
