# Supplementary material for: The Cheese Matrix Modulates the Immunomodulatory Properties of Propionibacterium freudenreichii CIRM-BIA 129 in Healthy Piglets
Source: Front Microbiol. 2018 Oct 29;9:2584. doi: 10.3389/fmicb.2018.02584 (PMC6215859; doi:10.3389/fmicb.2018.02584)
Supplement: Supplementary file 4 [file Table_1.docx]

|  |  | **Sequence (5′→3′)** | **Size (bp)** | **Accession no.** | **Reference** |
| --- | --- | --- | --- | --- | --- |
| **Propionibacteria**  5S  ssu  transcarboxylase | F  R | ATTCCATCGCCCTGAAGGA  TTGATCTGCGTCTTCTGGCC | 103 | AY861355.1 | Hervé et al., (2007) |
| **Family Bifidobacteriaceae**  **16S** | F  R | CGCGTCYGGTGTGAAAG  CCCCACATCCAGCATCCA | 244 | - | Delroisse et al.,(2006) |
| **Genus Lactobacillus**  **23S** | F  R | GCGGTGAAATTCCAAACG  GGGACCTTAACTGGTGAT | 216 | - | Hermann-Bank et al., (2013) |
| **Tbet** | F  R | TGGACCCAACTGTCAATTGCT  ACGGCTGGGAACGGGATA | 76 | NM_001315722.1 | Hernández et al., (2008) |
| **GATA3** | F  R | TGCGGGCTCTACCACAAAAT  TAACCCGAGTAAAATGTGC | 240 | DQ450901.1 | Muráni et al,. (2007) |
| **RORc** | F  R | GAAGTGGTGCTGGTCAGGAT  CGGGAGAAGTCAAAGATGGA | 140 | AK238874 | Young et al,. (2012) |
| **Foxp3** | F  R | GGTGCAGTCTCTGGAACAAC  GGTGCCAGTGGCTACAATAC | 148 | NM_001128438 | Zhu et al,. (2014) |
| **HPRT** | F  R | GGTGATAGATCCATTCCTATGACTGTAGA  TGAGAGATCATCTCCACCAATTACTT | 104 | U69731 | Ledger et al., 2004) |

**Table S1**. Specific primer sequences in order to target bacterial groups and species of high interest in the swine intestine as well as different transcriptional factors playing a key role for differentiation of immune cells.
